# An Ultrasonic Target Detection System Based on Piezoelectric Micromachined Ultrasonic Transducers

**DOI:** 10.3390/mi14030683

**Published:** 2023-03-19

**Authors:** Mingze Gao, Zhihao Tong, Zhipeng Wu, Liang Lou

**Affiliations:** 1 School of Microelectronics, Shanghai University, Shanghai 201800, China; 2 Shanghai Industrial μTechnology Research Institute, Shanghai 201899, China

**Keywords:** target detection system, ultrasonic sensor, Piezoelectric Micromachined Ultrasonic Transducers (PMUTs), robot control

## Abstract

In this paper, an ultrasonic target detection system based on Piezoelectric Micromachined Ultrasonic Transducers (PMUTs) is proposed, which consists of the PMUTs based ultrasonic sensor and the sensor system. Two pieces of 3 × 3 PMUTs arrays with the resonant frequency of 115 kHz are used as transmitter and receiver of the PMUTs-based ultrasonic sensor. Then, the sensor system can calculate the target’s position through the signal received by the above receiver. The static and dynamic performance of the proposed prototype system are characterized on black, white, and transparent targets. The experiment results demonstrated that the proposed system can detect targets of different colors, transparencies, and motion states. In the static experiments, the static location errors of the proposed system in the range of 200 mm to 320 mm are 0.51 mm, 0.50 mm and 0.53 mm, whereas the errors of a commercial laser sensor are 2.89 mm, 0.62 mm, and N\A. In the dynamic experiments, the experimental materials are the targets with thicknesses of 1 mm, 1.5 mm, 2 mm and 2.5 mm, respectively. The proposed system can detect the above targets with a maximum detection error of 4.00%. Meanwhile, the minimum resolution of the proposed system is about 0.5 mm. Finally, in the comprehensive experiments, the proposed system successfully guides a robotic manipulator to realize the detecting, grasping, and moving of a transparent target with 1 mm. This ultrasonic target detection system has demonstrated a cost-effective method to detect targets, especially transparent targets, which can be widely used in the detection and transfer of glass substrates in automated production lines.

## 1. Introduction

Target detection is widely used in the fields of autonomous driving [1,2], target tracking [3,4], obstacle avoidance [5,6], and so on. Among various target detection methods, the method of ultrasonic sensors based on Piezoelectric Micromachined Ultrasonic Transducers (PMUTs) has attracted much attention due to its small size, low cost, low power consumption, compatible with Complementary Metal Oxide Semiconductor (CMOS) process and simple system structure, which is an economical method to install on the robotic manipulator for detecting glass substrates in the automated production line.

One of the presence detection solutions for glass substrates is to use a set of through-beam photoelectric sensors. This method has the disadvantages of high cost, high installation accuracy requirements, and large interference from environment light. In order to reduce the interference of the above factors on the results, other sensors with better performance could be used as an alternative. Currently, the sensors used for target detection mainly include electromagnetic sensors, optical sensors, and ultrasonic sensors. Electromagnetic sensors are mostly used for dynamic detection because they are not affected by weather conditions and can obtain the relative position information of the detected targets through the Doppler property [7]. That said, detection results are prone to interference from metal objects such as conveyor belts and robotic manipulators. This means that the electromagnetic sensors are not suitable for industrial production lines [8]. Optical sensors include visual sensors [9,10,11], laser sensors [12,13,14], and infrared sensors [15,16]. Chen et al. proposed a stereo vision algorithm for the object pose estimation using point cloud data from multiple stereo vision systems [10]. Based on the visual sensor, this algorithm can accurately calculate the pose of targets, which can guide the subsequent movement and grasping of the robot. Han et al. proposed a control system of robotic manipulator grasping based on binocular visual sensors [17]. The Canny edge detection algorithm and inverse kinematics were used to solve the motion of the robotic manipulator, which improved the success rate of the robotic manipulator for grasping different targets. Visual sensors are too sensitive to environmental lighting. In addition, complex detection algorithms affect the real-time performance of these systems. In order to avoid the effect of environmental lighting, visual sensors can be replaced by laser sensors. Compared with visual sensors, laser sensors have longer detection distance and are more resistant to interference from to environmental lighting. Andry et al. used a 2D laser sensor fixed on a robotic manipulator to detect different targets, which proved the reliability of laser sensors under low contrast condition [18]. Although the laser sensors have excellent performance, higher accuracy comes at a significant expense. In addition, laser sensors cannot detect transparent targets. On the other hand, unlike environmental lighting, Kutila et al. pointed out that the performance of LiDAR (A device that uses laser and works like radar) decreased by 25% in harsh environments [12]. Compared to other optical sensors, infrared sensors play a more auxiliary role in the detection process. Lee et al. pointed out that infrared sensors can be used together with laser sensors in target tracking because they can provide effective nighttime visibility [15].

Compared with the above sensors, ultrasonic sensors are cheap and robust in a variety of environmental conditions. In autonomous driving, Jiménez et al. used ultrasonic sensors to replace laser sensors, which effectively reduced the cost [1]. Li et al. used a linear ultrasonic sensor array with an improved Kalman filter algorithm to achieve dynamic tracking of targets with different materials and shapes [3]. The above ultrasonic sensors are usually based on the bulk piezoelectric transducers. Although they have high output power, they are complex to manufacture and incompatible with the CMOS fabrication process [19]. PMUTs are generating sustained interest as a method to overcome the limitations of conventional ultrasonic sensors [20]. The PMUTs-based ultrasonic sensors are fabricated by Micro Electro Mechanical System (MEMS) technology, which makes the sensors low cost, physically small, and compatible with the CMOS fabrication process. These features make PMUTs-based ultrasonic sensors easy to integrate with other systems and widely used in gesture recognition [21], robot safety control [22], flow meters [23], and ranging [24].

In our previous work, an ultrasonic proximity-sensing skin based on PMUTs is proposed [25]. This skin can detect targets within 300 mm and 60 degrees in front, which contains two PMUTs-based receivers and a PMUTs-based transmitter. In the experiment, the skin is installed on the front end of the robotic manipulator to detect targets. As the robot manipulator moves toward the target point, if the target is within the dangerous distance, the robot manipulator will stop in an emergency to avoid collision. In this work, only a yellow transparent plate is used. The effects of color, transparency, and motion state of the targets are not investigated further.

In this paper, an ultrasonic target detection system based on PMUTs is proposed. Compared with our previous work, not only the effects of color, transparency, and the motion state of the targets on the detection results are investigated, but also the minimum resolution of the proposed system is investigated. The proposed system consists of the PMUTs-based ultrasonic sensor and the sensor system. The PMUTs-based ultrasonic sensor consists of a transmitter and a receiver, both of which are PMUTs arrays with a resonant frequency of 115 kHz. The received ultrasonic signals can be used to calculate the position of the targets through the sensor system. The static and dynamic performance of the proposed system are investigated, as well as a comparison with a commercial laser sensor. In the static experiment, the effects of targets with different colors and transparencies are investigated in the range of 150 to 250 mm. Black and white targets are used to verify the effect of color on the sensor. White and transparent targets are used to verify the effect of transparency on the sensor. In addition, the detection accuracy of the proposed system is also investigated. Compared with the laser sensor, the detection results of the PMUTs-based ultrasonic sensor is more stable, and the maximum error is 0.572 mm. In the dynamic experiment, the influence of the targets in motion is investigated to verify the effectiveness of the proposed system when targets move with the conveyor belt. Additionally, the minimum resolution of the system is measured to be about 0.5 mm. Finally, in order to investigate the communication function, the proposed system is combined with a robotic manipulator for the comprehensive experiment. In the comprehensive experiment, the UR3 robot manipulator is used to complete the detection, grasping, moving and reset of the 1 mm thickness target through information interaction under the control of the proposed ultrasonic target detection system. Based on the modularization and miniaturization of the proposed system, a glass substrate detection method with low cost, small size, and proper performance is realized.

## 2. Theory and Methods

### 2.1. The Method of Time of Flight

In the pulse echo method for ultrasonic sensors and laser sensors, Time of Flight (ToF) is the most widely used target detection method [26]. The transmitter emits a set of pulses, which are reflected to the receiver after encountering a target. Pulses can be absorbed, reflected, and transmitted on surfaces of different materials. Ignoring the influence of absorption, whether the target will be detected is related to the reflectivity and transmittance of the material. When the reflected echoes are received, there is a time delay between them and pulses. Therefore, the distance between the target and the sensor is related to the difference between the arrival time of the echoes and the emission time of the pulses, which is called the ToF. The distance *L* can be calculated by the following formula:

(1) $$L=\frac{vT}{2},$$

where *v* is the acoustic velocity in the air and *T* is the ToF. It is worth noting that major sources of data fluctuation can be found in additive noise affecting the acquired ultrasonic signal, shape distortion of the received echo, and dependence on temperature of the propagation velocity [27].

### 2.2. Reflectivity and Transmittance

The reflectivity and transmittance of ultrasonic waves are mainly related to the acoustic impedance. The acoustic impedance *Z* is expressed as:

(2) Z=ρc,

where *ρ* is the density of the material and *c* is the acoustic velocity in the materials. The reflectivity *r_u_* of ultrasonic waves is expressed as:

(3) $$r_{u}=\frac{Z_{2}-Z_{1}}{Z_{2}+Z_{1}},$$

where *Z*_1_ is the acoustic impedance of material 1 and *Z*_2_ is the acoustic impedance of material 2. The transmittance *t_u_* of ultrasonic waves is expressed as:

(4) $$t_{u}=\frac{2Z_{2}}{Z_{2}+Z_{1}}.$$

The above formulas show that the reflectivity and transmittance of ultrasonic waves are only related to the acoustic impedance. Since the acoustic impedance is an inherent constant of materials and is independent of color and transparency, the ultrasonic sensors are suitable for detecting the targets with different color and transparency. Similarly, the reflectance *r_l_* and transmittance *t_l_* of light waves are expressed as:

(5) $$r_{l}=\frac{n_{2}-n_{1}}{n_{2}+n_{1}},$$

(6) $$t_{l}=\frac{2n_{1}}{n_{2}+n_{1}},$$

where *n*_1_ is the refractive index of material 1 and *n*_2_ is the refractive index of material 2. The reflectivity and transmittance of light waves are related to the optical properties of materials. The optical properties of materials are affected by many factors, such as the colors and transparency. Therefore, even the same materials will show different results.

## 3. System Design

### 3.1. Structure of the Ultrasonic Sensor Based on PMUTs

**Figure 1:**
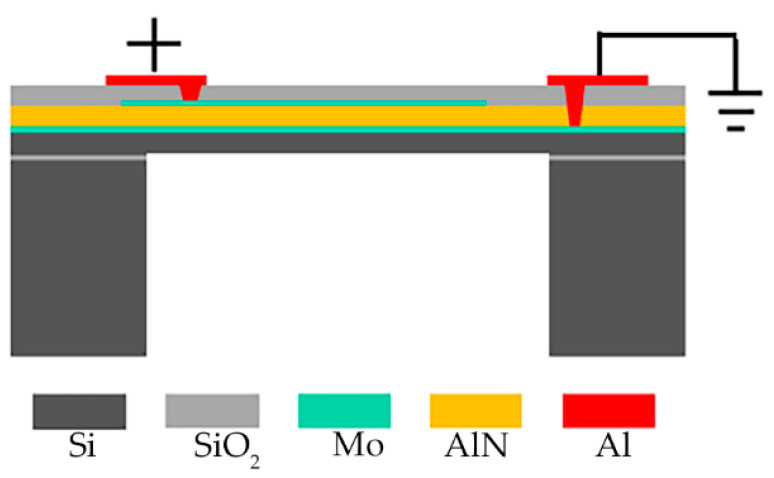
Cross-sectional view of PMUTs.

The PMUTs are fabricated on Silicon-On-Insulator (SOI) wafer [25]. As shown in Figure 1, the PMUTs diaphragm is comprised of five parts: a Si elastic layer, a piezoelectric Aluminum Nitride (AlN) layer, Molybdenum (Mo) layers for the top and bottom electrodes and oxide. The use of Mo as the electrode material can improve the structural properties of AlN thin films [28]. The mutual conversion of electrical energy and acoustic energy can be realized by this structure. When an AC signal is applied to the electrodes, the piezoelectric AlN layer will generate a transverse internal stress due to the piezoelectric effect, which drives the diaphragm to vibrate periodically and generate ultrasonic waves. On the contrary, the electrodes will detect electronic signals when the diaphragm is hit by ultrasonic waves. Geometric and performance parameters of the PMUTs array are investigated in our previous work. The first resonance frequency of the central element is about 115 kHz, the −3 dB bandwidth is 0.908 kHz, and the *Q* is 126.65. Besides, the displacement sensitivity of the central point is 13.2 nm/Vpp, which is measured through LDV under the sweep signal with the frequency from 1 kHz to 1 MHz [29].

**Figure 2:**
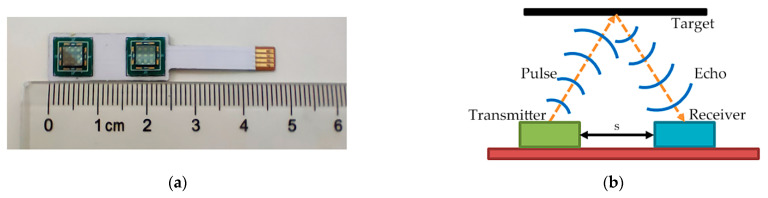
(**a**) The ultrasonic sensor based on PMUTs and (**b**) the target detection process of the sensor.

As shown in Figure 2a, the ultrasonic sensor based on PMUTs consists of two pieces of 3 × 3 PMUTs arrays as transmitter and receiver, respectively. The PMUTs arrays are integrated on a 0.5 mm thick Flexible Printed Circuit Board (FPCB) by wire bonding. The size of each piece of PMUTs array is 4 mm × 4 mm and the center distance between two pieces of PMUTs arrays is 15 mm. The size of the entire sensor is 10 mm × 47 mm × 1.7 mm. As shown in Figure 2b, the distance between the transmitter and the receiver is *s*. In the process of detection, when the target is placed parallel to the sensor, the path of the ultrasonic waves is an isosceles triangle. According to Equation (1), the distance *L* can be calculated by the following formula:

(7) $$L=\frac{\sqrt{v^{2}T^{2}-s^{2}}}{2}.$$

### 3.2. Design of the Sensor System

**Figure 3:**
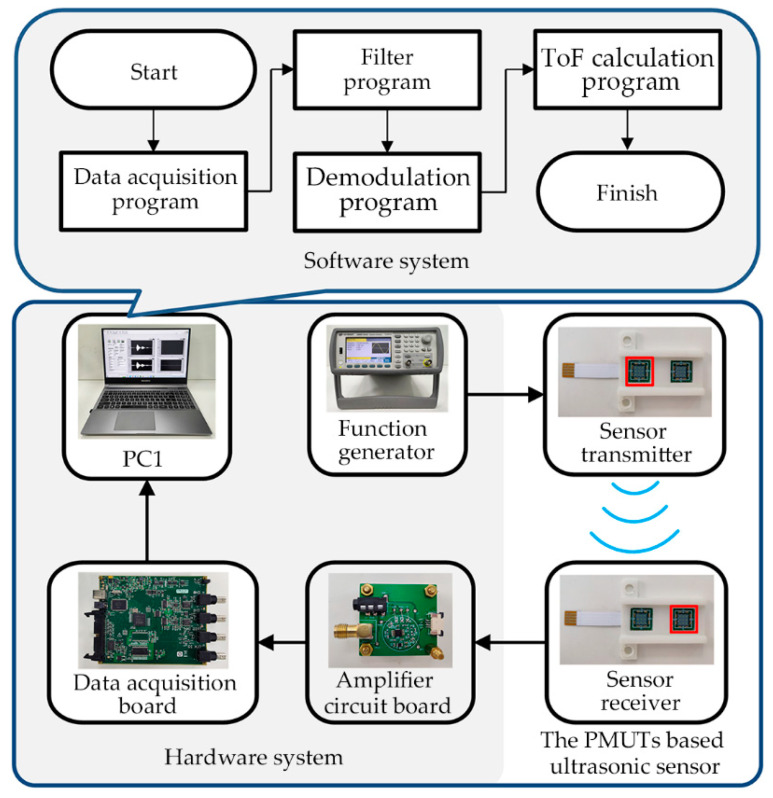
The hardware and software parts of the sensor system.

As shown in Figure 3, the sensor system consists of the hardware system and the software system. The hardware system includes the function generator, the amplifier circuit board, the data acquisition board, and the Personal Computer 1 (PC1). The function generator excites the transmitter of the PMUTs-based ultrasonic sensor to emit ultrasonic signals. Then, the received ultrasonic signals are amplified by the amplifier circuit board. The data acquisition board converts the analog signal into a digital signal. PC1 acts as a data processing center on which the software system runs. A typical control period of the hardware is as follows. First, the PMUTs-based ultrasonic sensor is excited by a burst signal (115 kHz, 5V_pp_, 10 sinusoidal). At the same time, the function generator sends a synchronization signal to the data acquisition board (MCC USB-2020). Then, the received ultrasonic signals are amplified by the amplifier circuit board. After acquisition by the data acquisition board, the ultrasonic signals are sent to PC1 (LabVIEW 2016).

**Figure 4:**
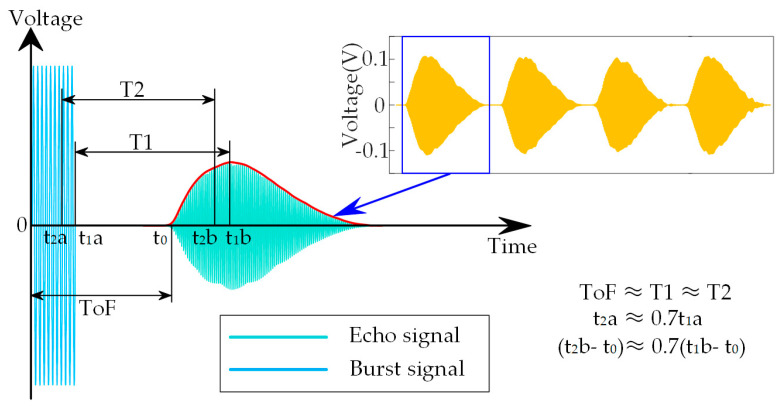
The consecutive echo signals and the envelope curve of the ultrasonic signals received.

The software system is used to calculate the position of the targets in real time. As shown in Figure 3, the software system includes the data acquisition program, the filter program, the demodulation program and the ToF calculation program. First, the received signals are converted into signal clusters through the data acquisition program with a sampling frequency of 1 MHz. Second, the wavelet filter and the Butterworth bandpass filter are used to remove noise in the filter program. The wavelet filter is used to filter out the noise near the resonant frequency, and the Butterworth bandpass filter is used to filter out the noise outside the resonant frequency. Then, the echo signals with high signal-to-noise ratio can be obtained. Third, the envelope of the ultrasonic echo signals is extracted by the Hilbert transform in the demodulation program. Finally, the ToF can be calculated through an improved peak detection algorithm. Compared with the traditional peak detection algorithm, noise has less influence on the improved algorithm. Therefore, the detection accuracy is higher. Figure 4 shows consecutive echo signals, and the envelope curve of the ultrasonic signals received. As shown in Figure 4, the actual ToF is the time from the start of the burst signal to the zero-crossing point of the echo signal (ToF). Since it is difficult to detect the zero-crossing point, in the traditional peak detection algorithm, the ToF is approximate to the time from the end of the burst signal to the peak value of the echo signal (T1). Due to the influence of noise, the peak value of the echo signal is still unstable in some situations. However, the ascent phase of the echo signal has the characteristic of monotonically increasing and is less affected by noise. Therefore, in the improved algorithm, the T1 is approximate to the time from 0.7 of the end of the burst signal to 0.7 of the peak value of the echo signal (T2). It should be noted that 0.7 is an empirical value. When the threshold is set to 0.7, more stable detection results can be obtained. In addition, when the echo signal is distorted or saturated, false detections caused by indistinct envelope peaks can be avoided using an improved peak detection algorithm. The detailed pseudocode of the improved peak detection algorithm is shown in Algorithm 1.
**Algorithm 1:** Calculate ToF from the envelope data.Input: Envelope data *D*; Threshold *h*; Waveform threshold *α*; Sampling frequency *s*;   Blind duration time *t*_m_; Pulse duration time *t*_j;_ Output: Time of Flight *ToF*;  1: Find peak *p* and peak index *i* in *D*;  2: **if**
*p > h*
**then**    Ture peak *P*_0_ = *p*;   **else** *P*_0_ = 0;   **end**  3: Threshold peak *P*_1_ = *αP*_0_;  4: Calculate the absolute value *A* between *D* and *P*_1_;  5: Find the data indices *B* where the *A* is the minimum value;  6: **if** *B* < *i* **then**    *B*_0_ = *B*;   **else** Find the minimum value of A again in the index 0 to B;   **end**  7: *ToF* = ((*B*_0_/*s) + t*_m_) − *αt*_j_;  8: **return** *ToF*;

### 3.3. Interaction with The Robotic Manipulator

**Figure 5:**
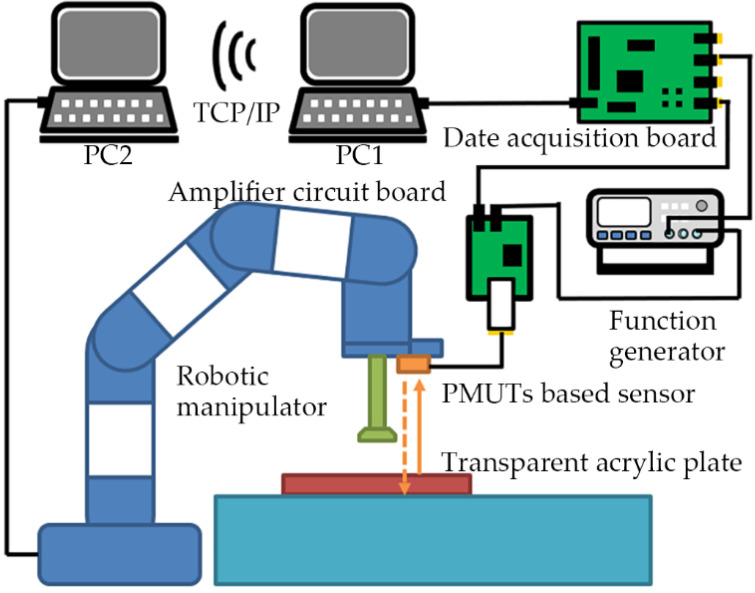
The ultrasonic target detection system in cooperation with a robotic manipulator.

The proposed system also contains interfaces to interact with other systems. As shown in Figure 5, the motion of the robotic manipulator can be controlled through communicating with the Robot Operating System (ROS). ROS runs on the Personal Computer 2 (PC2) based on the Linux system. PC2 communicates with the PC1 through TCP/IP. After PC1 sends a location message to PC2 through the ROS for LabVIEW, the MoveIt! controls the robotic manipulator (UR3) to move according to the solved optimal motion path [30]. At the same time, the motion states of the robotic manipulator can be displayed graphically in real time by Rviz. The ROS for LabVIEW is a set of VIs for communicating with ROS applications (VI is the smallest unit of execution in LabVIEW), developed at Tufts University by the Mechanical Engineering Department and the Center for Engineering Education and Outreach [31]. These VIs provide solutions for information interaction between LabVIEW and ROS. The MoveIt! is an open-source robotic manipulation platform that allows the development complex manipulation applications using ROS [32].

## 4. Experiment Setup

The characteristics of the proposed system are investigated by static experiments, dynamic experiments (including the comparison with a commercial laser sensor), and comprehensive experiments. Based on the laboratory environment, the acrylic plates are selected as experimental materials.

**Figure 6:**
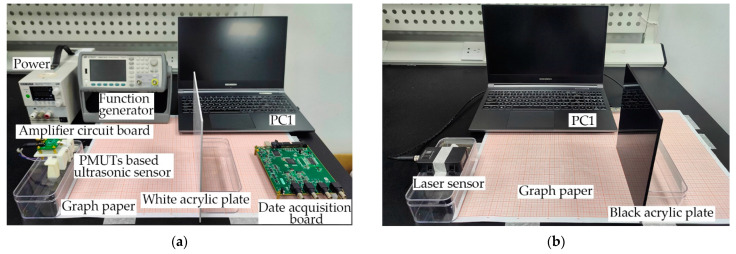
Static experimental setup of (**a**) the PMUTs-based ultrasonic sensor and (**b**) the laser sensor.

In the static experiments, the effects of color and transparency on the detection results are investigated. The experimental setup of the PMUTs-based ultrasonic sensor is shown in Figure 6a. The PMUTs-based ultrasonic sensor is placed on one side of the coordinate paper with an accuracy of 1 mm. The acrylic plates are placed on the other side. The thicknesses of black, white, and transparent acrylic plates are all 5 mm. Black and white acrylic plates are used as experimental targets to compare the effect of color, whereas white and transparent acrylic plates are used as experimental targets to compare the effect of transparency on experimental results. The acrylic plates are moved from 200 mm to 320 mm in 5 mm steps. Similarly, the experimental setup of the laser sensor (MyAntenna L1s-40) is shown in Figure 6b. The relevant parameters of the laser sensor are shown in Table 1.

**Figure 7:**
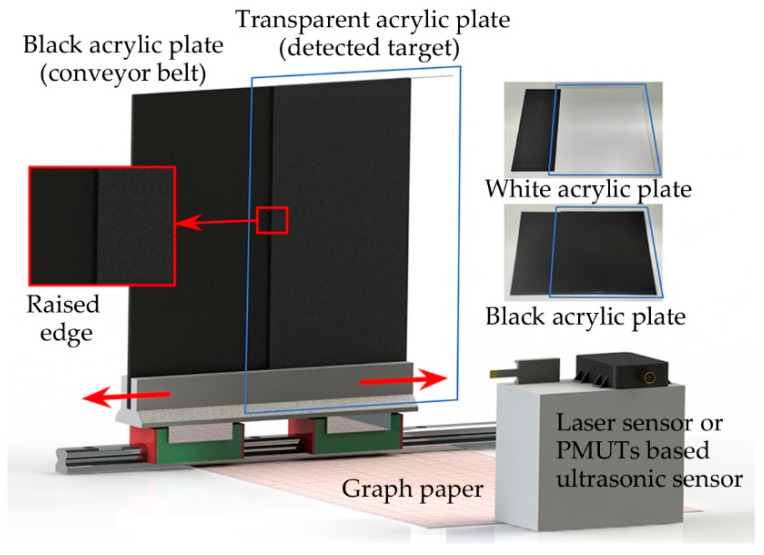
Dynamic experimental setup.

In the dynamic experiments, the effects of targets in motion on the detection results are investigated by simulating the motion of the targets on the conveyor belt. As shown in Figure 7, based on the laboratory environment, a black acrylic plate is used as the bottom plate instead of the conveyor belt, and a black, white, or transparent acrylic plate is placed on the front side. The stacked acrylic plates are placed on a linear guide. The linear guide is placed parallel to the sensors, which allows the acrylic plates to move horizontally relative to the sensors. The thicknesses of the three kinds of acrylic plates are about 1 mm, 1.5 mm, 2 mm, and 2.5 mm. Measured by a vernier caliper, for black acrylic plates, the actual thicknesses are 1.08 mm, 1.69 mm, 1.84 mm, and 2.42 mm. For white acrylic plates, the actual thicknesses are 1.00 mm, 1.73 mm, 1.80 mm, and 2.57 mm. For transparent acrylic plates, the actual thicknesses are 0.97 mm, 1.39 mm, 1.86 mm, and 2.31 mm. With the movement of the linear guide, the detection point of the sensors is transferred from the upper surface of the black bottom plate (the lower surface of the targets) to the upper surface of the targets. Then, the thicknesses of the acrylic plates can be detected by the difference between the detection results of the sensors. In addition, the minimum resolution of the proposed system is discussed by using the transparent acrylic plates with small thickness.

**Figure 8:**
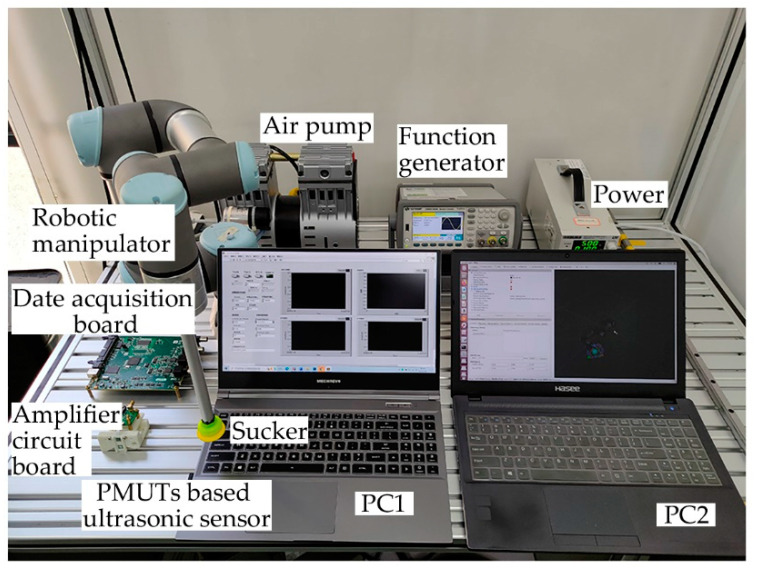
The devices of the comprehensive experiment.

In order to investigate the feasibility of the proposed system to cooperate with other systems, the comprehensive experiments are designed by combining the proposed system and a robotic manipulator. The working environment in an automated production line has been simulated in the laboratory. A transparent acrylic plate with a thickness of 1 mm needs to be grabbed by a robotic manipulator from the starting point and placed to the target point according to a fixed path. The devices of the comprehensive experiment are shown in Figure 8. The experimental process is as follows: initially, the robotic manipulator stops above the starting point; the PMUTs-based ultrasonic sensor placed at the end of the robotic manipulator detects surroundings in real time and returns distance values to PC1; when transparent acrylic plate is detected on the starting point, the distance values will be changed; then the robotic manipulator is controlled to grab the transparent acrylic plate and move to the target point at a speed of 0.1 m/s.

## 5. Results

### 5.1. Static Experiment Results

**Figure 9:**
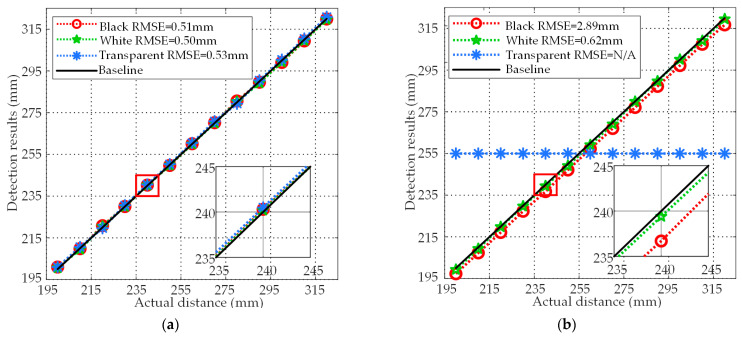
(**a**) Results of the PMUTs-based ultrasonic sensor and (**b**) the laser sensor in the static experiments.

Usually, the detection result cannot be determined by a single result. It is a common to take the average value of multiple groups of results. In order to get a comprehensive performance, the detection results are evaluated by Root Mean Square Error (RMSE). The RMSE is the error between the measurements and the true values which represents the accuracy of the detection [33]. The formula of RMSE is as follows:

(8) $$\mathrm{RMSE}=\sqrt{\frac{\sum_{i=1}^{n}{\left(f_{i}-y_{i}\right)}^{2}}{n}},$$

where *y_i_* are the true distance values, which are measured by coordinate paper. The *f_i_* are the average values of detection results under this distance. According to the data, the experiment results are shown in Figure 9. For black, white, and transparent acrylic plates, the RMSEs of the PMUTs based ultrasonic sensor are 0.51 mm, 0.50 mm, and 0.53 mm, and the RMSEs of the laser sensor are 2.89 mm, 0.62 mm, and not applicable. For the PMUTs based ultrasonic sensor, the maximum difference between different RMSEs is 0.03 mm, which means that colors and transparency have little effect on the detection results. Corresponding to the RMSEs, the detection curves of the PMUTs-based ultrasonic sensor almost overlap. On the other hand, for the laser sensor, the detection curve of the black acrylic plates is obviously shifted from the baseline. This is caused by the weakening of the received signals strength due to the absorption of light waves by the black targets. For transparent acrylic plates, the RMSE is not applicable since the laser sensor cannot detect the results on transparent targets. This is because most of light waves are transmitted due to the high transmittance. As shown in Table 2, 80.321% of the light waves penetrated the transparent acrylic plates. Only 19.679% of the light waves were reflected, which is not enough to be used to calculate the targets position. The static experiments show that colors and transparency of the targets cannot affect the detection results of the proposed system. In contrast, the laser sensor cannot accurately detect black and transparent targets because light can be absorbed by black targets and penetrate transparent targets.

### 5.2. Dynamic Experiment Results

**Figure 10:**
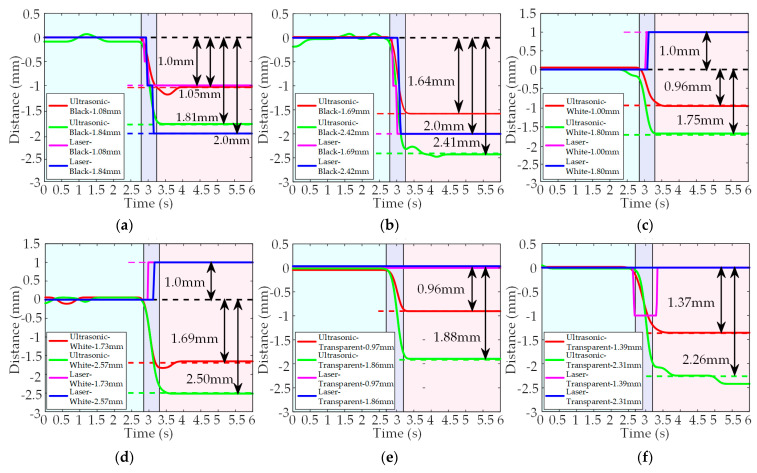
Detection results of the PMUTs-based ultrasonic sensor and the laser sensor when using black acrylic plates with thicknesses of 1 mm and 2 mm (**a**), 1.5 mm and 2.5 mm (**b**), white acrylic plates with thicknesses of 1 mm and 2 mm (**c**), 1.5 mm and 2.5 mm (**d**), transparent acrylic plates with thicknesses of 1 mm and 2 mm (**e**), 1.5 mm and 2.5 mm (**f**) in the dynamic experiments.

In the dynamic experiments, as shown in Figure 10, the changes in detection curves represent the thicknesses of the targets. When using the black acrylic plates as the detected targets, the detection results are shown in Figure 10a,b. For the PMUTs-based ultrasonic sensor, the difference between the detection values and the actual thicknesses of the black acrylic plates are 0.03 mm, 0.05 mm, 0.03 mm and 0.01 mm, respectively, and the errors are 2.78%, 2.96%, 1.63% and 0.41%, respectively. For the laser sensor, since the minimum resolution is 1 mm, the acrylic plates with thicknesses of 1.5 mm and 2.5 mm cannot be distinguished. Therefore, the detection values are 1.0.mm, 2.0 mm, 2.0 mm, and 2.0 mm. When the black acrylic plates are replaced by the white acrylic plates, the detection results are shown in Figure 10c,d. For the PMUTs-based ultrasonic sensor, the difference between the detection values and the actual thicknesses of the white acrylic plates are 0.04 mm, 0.04 mm, 0.05 mm and 0.07 mm, while the errors are 4.00%, 2.31%, 2.78% and 2.72%, respectively. In contrast, the detection values of the laser sensor increase as the thicknesses increases. Because the changes in thicknesses are less than the RMSE of the black acrylic plates as the bottom plate, the detection values do not decrease, but increase. When using the transparent acrylic plates as the detected targets, the detection results are shown in Figure 10e,f. For the PMUTs-based ultrasonic sensor, the difference between the detection values and the actual thicknesses of the transparent acrylic plates by 0.01 mm, 0.02 mm, 0.02 mm and 0.05 mm, and the detection errors are 1.03%, 1.44%, 1.08% and 2.16%, respectively. For the laser sensor, the detection values do not change significantly. The fluctuation of 1 mm might be caused by the interference of the environmental noise. The light waves penetrate the transparent acrylic plates and only the light waves reflected from the black acrylic plates are detected by the laser sensor. The minimum resolution of the PMUTs-based ultrasonic sensor is further investigated.

**Figure 11:**
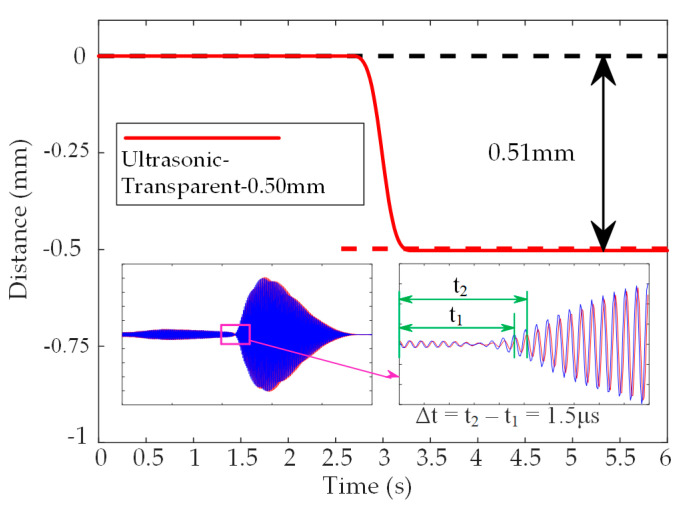
Detection results of the PMUTs-based ultrasonic sensor when using transparent acrylic plate with thicknesses of 0.501 mm and the changes of the detection signals.

As shown in Figure 11, when using a transparent acrylic plate with an actual thickness of 0.50 mm, the detection value of the proposed system is 0.51 mm. The dynamic experiments demonstrate that the proposed system can detect targets with high precision. Even if the targets are in motion, the detection results are not affected by the color or transparency. In the experiments, the maximum detection error of the proposed system is 4.00% for targets with the thicknesses of 1 mm, 1.5 mm, 2 mm and 2.5 mm. At the same time, the minimum resolution is about 0.5 mm. In contrast, the laser sensor only has a minimum resolution of 1 mm. Therefore, the acrylic plates with thicknesses of 1.5 mm and 2.5 mm cannot be detected. In addition, the laser sensor cannot detect transparent targets.

### 5.3. Comprehensive Experiment Results

**Figure 12:**
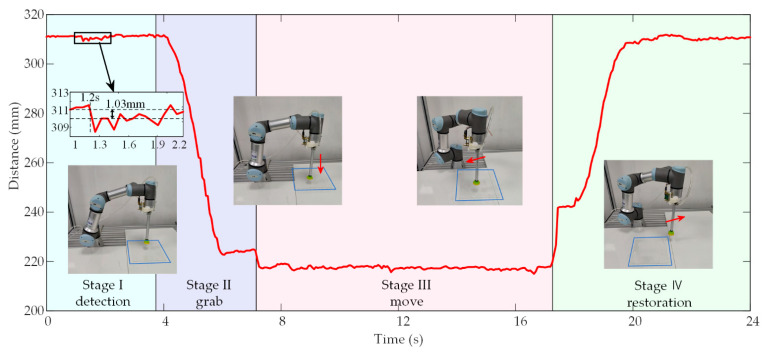
Detection results of the proposed system in comprehensive experiments.

The comprehensive experimental results are shown in Figure 12. The curve represents the variation of distance values in the whole comprehensive experiment. The result is divided into four stages as follows. Stage I (0–3.8 s, detection): the PMUTs-based ultrasonic sensor detects distance values in real time when placed at 311.36 mm high. As shown in the 1.2 s, when the transparent acrylic plate with a thickness of 1 mm is placed to the starting point, the distance value changes to 310.33 mm. This means that the transparent acrylic plate is detected. Stage II (3.8–7.2 s, grab): the robotic manipulator moves down and grabs the transparent acrylic plate, causing the detection distance values to drop significantly. Stage III (7.2–17.2 s, move): the transparent acrylic plate is moved by the robotic manipulator and placed at the target point. Stage Ⅳ (17.2–24.0 s, restoration): the robotic manipulator moves up and returns to the starting point.

As shown in Figure 12, the distance values fluctuate in the whole process. This may be caused for two reasons. First, when the robotic manipulator is stationary, the fluctuations are mainly related to environmental noise. Second, when the robotic manipulator is in motion, the fluctuations are mainly related to the vibrations of the motors. At the same time, when the PMUTs-based ultrasonic sensor is fixed on the robotic manipulator, small vibrations of the robotic manipulator in motion can be detected by the above sensor. These signals will be coupled into the detection signals. The response time of the whole system is about 330 ms, including the burst period of 50 ms and the 89 ms for calculating the optimal path and controlling the robotic manipulator. According to user manual, the robot manipulator has a response time of 159 ms. The above factors account for 90.303% of the total response time, the rest of response time may come from the transmission delay of the local area network. The comprehensive experimental results show that the proposed ultrasonic target detection system can successfully detect transparent targets and guide the robotic manipulator to complete practical production tasks such as grasping and moving.

## 6. Conclusions

In this paper, an ultrasonic target detection system based on PMUTs is proposed, which can obtain the precise position of the targets. This proposed system consists of the PMUTs-based ultrasonic sensor and the sensor system. The sensor system can interact with the robotic manipulator through TCP/IP. The characteristics of the proposed system under static and dynamic conditions are characterized, and a comparison is made with a commercial laser sensor. Even with different colors, transparencies and motion states, the proposed system can accurately detect the position of the targets. In the static experiments, the static location errors of the proposed system are 0.51 mm, 0.50 mm and 0.53 mm for black, white, and transparent targets, respectively, in the range of 200 to 320 mm. In contrast, the RMSEs of the laser sensor are 2.89 mm, 0.62 mm, and not applicable. Since light waves can be absorbed by black targets, there is a significant difference in the accuracy of the two sensors. In addition, the detection results of transparent targets cannot be compared. In the dynamic experiments, for the targets with thickness of 1 mm, 1.5 mm, 2 mm and 2.5 mm, the maximum detection error of the proposed system is 4.00%. In contrast, the laser sensor cannot distinguish targets with thicknesses of 1.5 mm and 2.5 mm due to the minimum resolution of 1 mm. Unlike the laser sensor, the minimum resolution of the proposed system is about 0.5 mm. Besides, the comprehensive experiments verify the feasibility of the proposed system in cooperation with the robotic manipulator. In the comprehensive experiments, the system is required to detect a transparent target with a thickness of 1 mm and control the robotic manipulator to complete the subsequent grasping and moving operations. The experimental results demonstrate that the proposed system successfully accomplishes the specified task, which shows the potential in target detection especially for transparent targets. With the further development of technology, the glass substrates can be more widely used in smart phones and smart screens. Therefore, the thicknesses of the glass substrates will be further reduced. In order to improve the scope of application of the proposed system, improving the detection algorithm to improve the detection accuracy is the focus of the next work. On the other hand, the proposed system is only prototype systems. How to improve the integration of the hardware part in the sensor system is also an aspect that cannot be ignored.

## Figures and Tables

**Table 1:** The Relevant Parameters of The Laser Sensor.

Related Parameters	Values
Detection range (m)	0.05~40
Resolution (mm)	1
Repeatability (mm)	±1
Measurement rate (Hz)	5 (Continuous measurement mode) 20 (Fast measurement mode)
Price ($)	50

**Table 2:** Reflectivity and Transmittance of Ultrasonic and Light Waves.

	Air	Acrylic Plates	Ultrasonic Waves	Light Waves
Density(kg/m^3^)	1.210	1.200 × 10^3^		
Speed of Sound(m/s)	3.420 × 10^3^	2.700 × 10^3^		
Acoustic Impedance (Ns/m^3^)	4.150 × 10^3^	3.240 × 10^3^		
Refractive Index	1.000	1.490		
Reflectivity			99.981%	19.679%
Transmittance			0.026%	80.321%
